# The usefulness of 3D printed heart models for medical student education in congenital heart disease

**DOI:** 10.1186/s12909-021-02917-z

**Published:** 2021-09-08

**Authors:** Clement Karsenty, Aitor Guitarte, Yves Dulac, Jerome Briot, Sebastien Hascoet, Remi Vincent, Benoit Delepaul, Paul Vignaud, Camelia Djeddai, Khaled Hadeed, Philippe Acar

**Affiliations:** 1 Pediatric cardiology unit, Children Hospital, CHU Toulouse, 330 Avenue de Grande Bretagne TSA 70034, 31059 Toulouse cedex 9, France; 2 Institut Des Maladies Métaboliques Et Cardiovasculaires, Université de Toulouse, INSERM U1048, I2MC, 1, Avenue Jean Poulhès-BP84225, Toulouse, France; 3 Department of Pediatric and Adult Congenital Heart Diseases, Marie Lannelongue Hospital, Groupe Hospitalier Saint Joseph Reference Center of Complex Congenital Heart Diseases M3C, Le Plessis Robinson, France

**Keywords:** Congenital heart disease, 3D printing model, Teaching, Undergraduate medical students

## Abstract

**Background:**

Three-dimensional (3D) printing technology enables the translation of 2-dimensional (2D) medical imaging into a physical replica of a patient’s individual anatomy and may enhance the understanding of congenital heart defects (CHD). We aimed to evaluate the usefulness of a spectrum of 3D-printed models in teaching CHD to medical students.

**Results:**

We performed a prospective, randomized educational procedure to teach fifth year medical students four CHDs (atrial septal defect (ASD, *n* = 74), ventricular septal defect (VSD, *n* = 50), coarctation of aorta (CoA, *n* = 118) and tetralogy of Fallot (ToF, *n* = 105)). Students were randomized into printing groups or control groups. All students received the same 20 min lecture with projected digital 2D images. The printing groups also manipulated 3D printed models during the lecture. Both groups answered an objective survey (Multiple-choice questionnaire) twice, pre- and post-test, and completed a post-lecture subjective survey.

Three hundred forty-seven students were included and both teaching groups for each CHD were comparable in age, sex and pre-test score. Overall, objective knowledge improved after the lecture and was higher in the printing group compared to the control group (16.3 ± 2.6 vs 14.8 ± 2.8 out of 20, *p* < 0.0001). Similar results were observed for each CHD (*p* = 0.0001 ASD group; *p* = 0.002 VSD group; *p* = 0.0005 CoA group; *p* = 0.003 ToF group). Students’ opinion of their understanding of CHDs was higher in the printing group compared to the control group (respectively 4.2 ± 0.5 vs 3.8 ± 0.4 out of 5, *p* < 0.0001).

**Conclusion:**

The use of 3D printed models in CHD lectures improve both objective knowledge and learner satisfaction for medical students. The practice should be mainstreamed.

**Supplementary Information:**

The online version contains supplementary material available at 10.1186/s12909-021-02917-z.

## Introduction

Congenital heart defects (CHD) are a leading cause of morbidity in pediatric patients and is an emerging field in adult medicine. Therefore, the anatomy and pathophysiology of CHD is an integral and increasingly important part of medical education [1]. Two-dimensional (2D) representations are the mainstream method of teaching CHD. The ability to translate these 2D images into 3-dimensional (3D) representations of the defect is critical to the understanding, diagnosis, and management of underlying disease. Individuals vary in their ability to deduce 3D spatial relationships from 2D imaging [2]. Since the first stereolithography technique used by Charles Hull in 1983, the method of transforming digital models into physical objects has been one of the main disruptive technologies in the first two decades of the twenty-first century. In healthcare, 3D printing is being widely adopted in many areas, from creating bioprosthetics, to improving surgical planning and as an educational tool [3–6]. 3D models provide a unique support for the comprehension of simple to more complex cardiac malformations. Compared to other tissue engineering scaffolds, 3D printing has the advantages of relatively low production costs and accurate generation of an anatomical structure in a short time [7]. In order to create 3D printed heart and blood vessel models, multiple imaging techniques might be used to obtain a volumetric representation of the heart, such as computed tomography (CT), 3D echocardiography and cardiac magnetic resonance imaging [8].

Few studies have evaluated the usefulness of 3D printed heart models to teach CHD to medical students, and most of the research has focused on learners satisfaction or has often analysed a single CHD with a small student sample [9–12]. Moreover, studies have reported heterogeneous conclusions with regards to learners’ knowledge acquisition after teaching with 3D printed heart models, regardless of the disease complexity.

We aimed to evaluate the usefulness of 3D printed models in teaching medical students four CHD, using a randomized controlled trial design.

## Method

### Creating 3D printed models

This study was approved by the Institutional Review Board at the Toulouse University Hospital Affiliated with Toulouse University Paul Sabatier School of Medicine in the form of modified teaching method study. All methods were performed in accordance with the relevant guidelines and regulations.

**Fig. 1:**
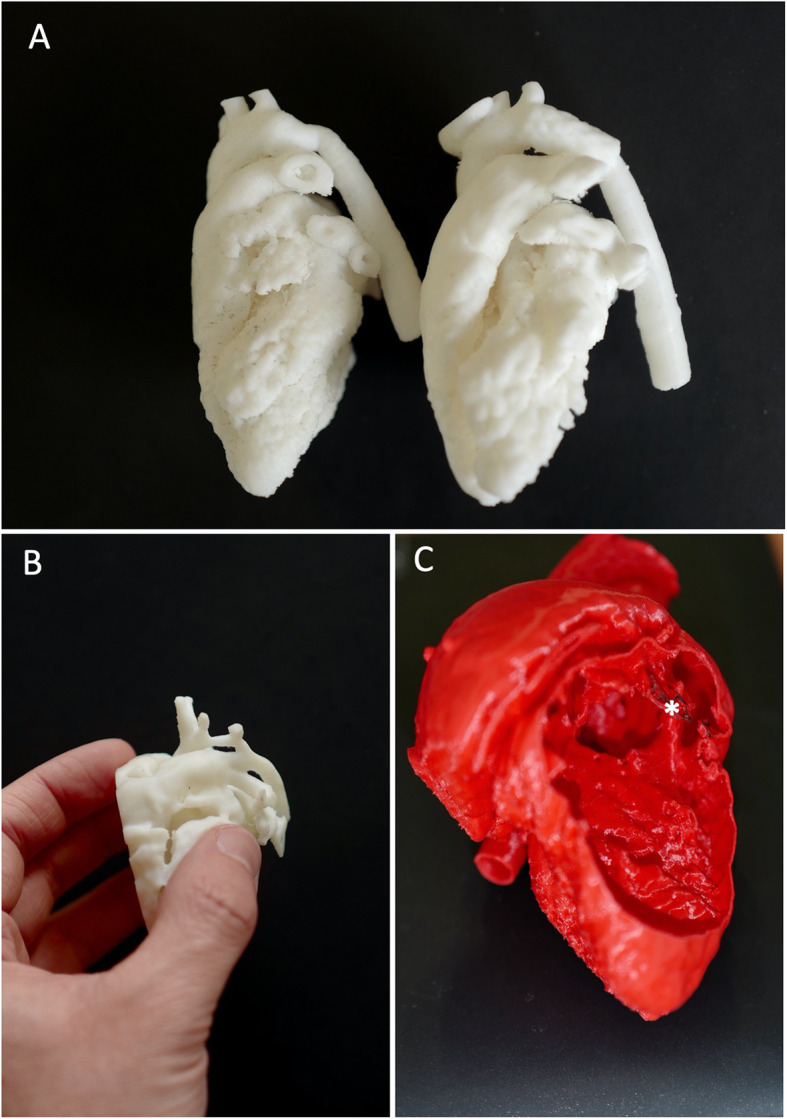
Examples of 3D printed heart models. Panel **A** *left =* isthmic aortic coarctation, *right* = stent-repaired of the same patient. Panel **B** neonatal aortic hypoplasia with patent ductus arteriosus. Panel **C** tetralogy of Fallot; asterisk is pointed the anterior deviation of the conal septum

We conducted a retrospective search for extractable DICOM files of cardiac CT and 3D echocardiography performed in our hospital over the past year in routine follow-up of CHD. Based on the quality, after anonymization, we selected four CT exams of coarctation of the aorta (CoA): one neonatal case, one infant, one stent-repaired CoA, and one aortic hypoplasia; four CT exams of ventricular septal defect (VSD): one perimembranous VSD, one inlet VSD, one outlet VSD without malalignment, one outlet VSD with malalignment; two CT exams and one 3D echocardiography of atrial septal defect (ASD): two different ostium secundum ASDs and one sinus venosus ASD; and two CT exams of Tetralogy of Fallot (ToF) (Fig. 1 and S1 ToF, S2 neonatal aortic hypoplasia, S3 VSD: 3D PDF). These four CHDs are among the most common and are included in the French medical program. We used Mimics and 3-Matic (Materialise HQ, Leuven, Belgium) software for segmentation and to generate the final real scale 3D virtual model exported as an STL file. STL files were finally printed with a Stream 20 pro printer (Volumic, France) and biodegradable polylactic acid (PLA) filament. For the ASD model from echocardiography data, image was acquired by 3D transesophageal echocardiography in 3D zoom mode using the EPIQ system (version 7C, Philips Medical Systems, Andover, MA) and an X8-2t phased array transducer. All models were printed in duplicate for a total of 26 models. The mean period time needed to segmentate models was around 15 min for ASD and CoA, 30 min for VSD and 60 min for ToF. The mean period time to print the models were 30 min for ASD, and 150 min for ToF, CoA and VSD.

### Study population and intervention

Fifth year medical students from the Paul Sabatier University of Toulouse faculty of medicine were anonymously included. In France, medical degree is validated at the end of the 6th year corresponding to a MBBS. The paediatric module was shared with gynaecology, orthopaedic, and rheumatology and clinical practice was provided during 1 month after a 1 month of full lecture. All students attended lectures on CHD as part of the standard academic training. Different students attended additional 20-min lectures for one of the following CHD: CoA, VSD, ASD and ToF. Lectures were held on separate days by the same teacher and so each learner attended only one lecture for one of the four different CHD.

For each 20-min CHD lecture, students were randomized into two groups; one was the intervention group (printing) and the other a control group (control). Both groups attended the same lecture in two separate sessions which consisted of standard slides and projected two-dimensional models on anatomy, physiopathology and the diagnosis and management of CHD. After an initial description of each printed model, students in the printing group had the possibility during all the lecture to freely analyze and manipulate 3D printed models for the CHD presented by sharing models and passing them around during the lectures. Students were also free to ask question about the models during the lecture.

### Acquisition evaluation

To evaluate knowledge acquisition, each student answered the same multiple-choice test twice, pre- and post-lecture, on the respective CHD with a maximal score of 20 points corresponding to 20 true or false questions. Questionnaires assessed knowledge acquisition on anatomy, pathophysiology and overall management. Questionnaires were made by PA, CK, and AG and met the faculty’s end-of-year exam validation criteria. In the post-lecture test, we also included three more 5-point Likert scale (1 to 5: 1 = strongly disagree, 5 = strongly agree) questions to assess self-rating of acquired general knowledge and knowledge on the diagnosis and treatment of CHD. The participants in the printing group were also asked to rate their satisfaction with the 3D printed models as a teaching tool, and to use a 5-point Likert scale to indicate whether this helped them to understand the presented CHD. An example of the post-test questionnaire including the objective and subjective questionnaire is provided (S4 and S5: supplementary method).

### Analysis

Quantitative variables are expressed as mean ± standard deviation (SD). Normally distributed continuous variables were compared with t tests and non-normally distributed variables were compared with Mann-Whitney tests. Normality was assessed by the Shapiro-Wilk normality test. Changes in subjective survey scores were compared with t tests or Mann-Whitney tests in terms of the normality distribution. Knowledge ratings from the ‘pre-’ and ‘post’-intervention survey were analysed using a paired test for each CHD or a Wilcoxon matched-pairs signed rank test if the distribution was not normal. Increases in scores were assessed by subtracting the pre-test score from the post-test score. The printing group was then compared to the control group with a t test.

*P*-values < 0.05 were considered statistically significant. Statistical analysis was carried out using GraphPad Prism 9 (GraphPad Software, Inc., San Diego, CA).

## Results

We enrolled 347 students including 74 in the ASD group (55.4% in the printing group), 50 in the VSD group (50% in the printing group), 118 in the CoA group (55% in the printing group), and 105 in the ToF group (49.5% in the printing group). The model/student ratio was 1/7 for ASD, 1/3 for VSD, 1/8 for CoA, and 1/13 for ToF.

The two teaching groups for each CHD were comparable in age, gender and pre-test score (Table 1).

**Table 1:** Baseline characteristics of students in the control and printing groups

Group	Control	Printing	***p***-value
**ASD (*n*** **= 74)**	33	41	
Female (%)	79	73	0.6
Age (years)	21.8 ± 1.2	21.7 ± 0.8	0.53
Pre-test score (/20)	10.5 ± 0.4	11.1 ± 0.3	0.21
**VSD (*n*** **= 50)**	25	25	
Female (%)	78	72	0.62
Age (years)	22.2 ± 2.2	21.8 ± 0.9	0.86
Pre-test score (/20)	9.8 ± 0.5	10.0 ± 0.5	0.77
**CoA (*n*** **= 118)**	53	65	
Female (%)	60	66	0.56
Age (years)	22.8 ± 1.3	22.8 ± 2.0	0.82
Pre-test score (/20)	12.8 ± 0.3	12.9 ± 0.3	0.74
**ToF (*n*** **= 105)**	53	52	
Female (%)	69	77	0.49
Age (years)	22.8 ± 1.3	22.8 ± 2.0	0.82
Pre-test score (/20)	11.8 ± 0.3	11.7 ± 0.3	0.89

*ASD* Atrial septal defect, *CoA* Coarctation of the aorta, *CHD* Congenital heart defects, *ToF* Tetralogy of Fallot, *VSD* Ventricular septal defect

**Fig. 2:**
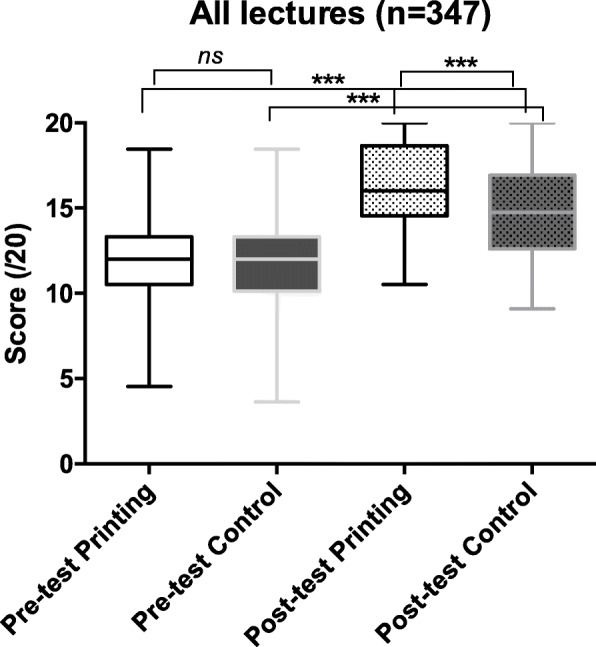
Pre- and post-lecture test results for the control and printing group including all CHD, ***p < 0.001. CHD: Congenital heart defects

Overall, there was no difference in the pre-lecture tests between printing and control groups, with a median global score of 11.8 ± 2.4 out of a maximal score of 20 for the control group and 11.6 ± 2.5 out of 20 for the printing group (*p* = 0.46) (Fig. 2). Knowledge improved in both groups after the lecture but was higher in the printing group than the control group (16.3 ± 2.6 vs 14.8 ± 2.8 out of 20, *p* < 0.0001). The increase in score was higher in the printing group than the control group (4.5 ± 0.4 vs 3.2 ± 0.5, *p* = 0.001).

**Fig. 3:**
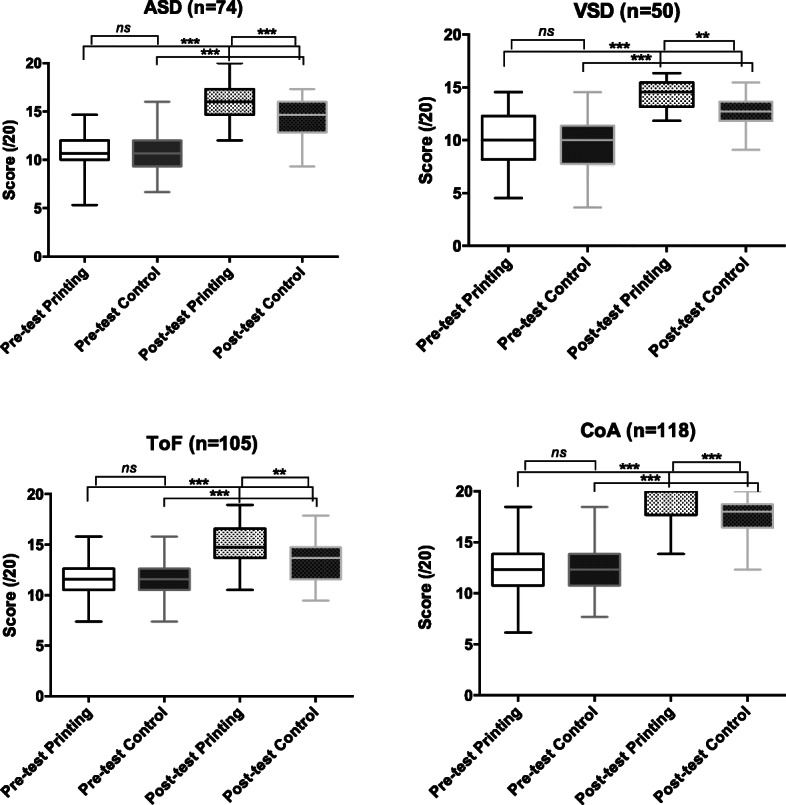
Pre- and post-lecture test results for the control and printing group according to the CHD (ASD, VSD, CoA, and ToF). **0.01 < *p* < 0.001, ***p < 0.001. ASD: Atrial septal defect; CoA: Coarctation of the aorta; CHD: Congenital heart defects; ToF: Tetralogy of Fallot; VSD: Ventricular septal defect

Similarly, for each CHD group, no difference in the baseline objective test scores was observed, and all groups improved after the lecture. The post-lecture scores were significantly higher for the printing group than for the control group, regardless of the CHD (*p* = 0.0001 in the ASD group; *p* = 0.002 in the VSD group; *p* = 0.0005 in the CoA group; *p* = 0.003 in the ToF group), (Fig. 3). The increase in score was also higher for each CHD in the printing group (Table 2).

**Table 2:** Pre- and post-test increase in score (out of 20) in the control and printing groups for each CHD

Group	Control	Printing	***p***-value
**ASD**	3.5 ± 0.4	4.9 ± 0.3	0.01
**VSD**	2.7 ± 0.4	4.1 ± 0.5	0.02
**CoA**	4.6 ± 0.4	5.8 ± 0.4	0.03
**ToF**	1.5 ± 0.3	3.0 ± 0.2	< 0.0001

*ASD* Atrial septal defect, *CoA* Coarctation of the aorta, *CHD* Congenital heart defects, *ToF* Tetralogy of Fallot, *VSD* Ventricular septal defect

**Fig. 4:**
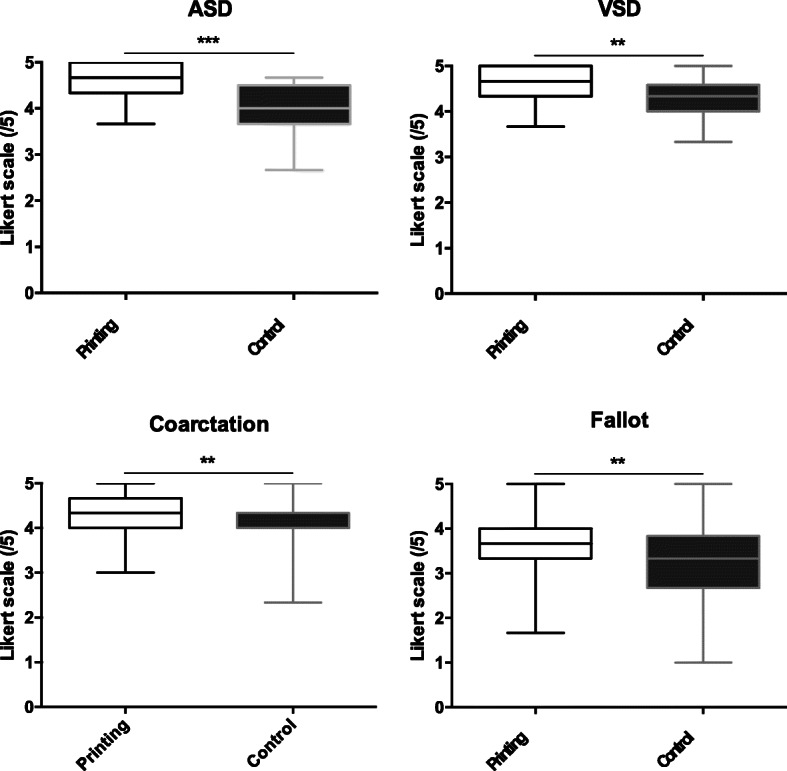
Students’ self-reported understanding of CHDs, their diagnostic modalities and treatment options: boxplot of means of three 5-point Likert scale items according to the CHD (ASD, VSD, CoA, and ToF). ASD: Atrial septal defect; CoA: Coarctation of the aorta; CHD: Congenital heart defects; ToF: Tetralogy of Fallot; VSD: Ventricular septal defect

In the post-lecture subjective 5-point Likert scale evaluation, the students learning through 3D printed models scored higher in their self-reported understanding of the CHDs, their diagnostic modalities and treatment options (Fig. 4). For all CHDs combined, the mean 5-point Likert scale was 3.8 ± 0.4 out of 5 for control groups versus 4.2 ± 0.5 for printing groups (*p* < 0.0001).

**Fig. 5:**
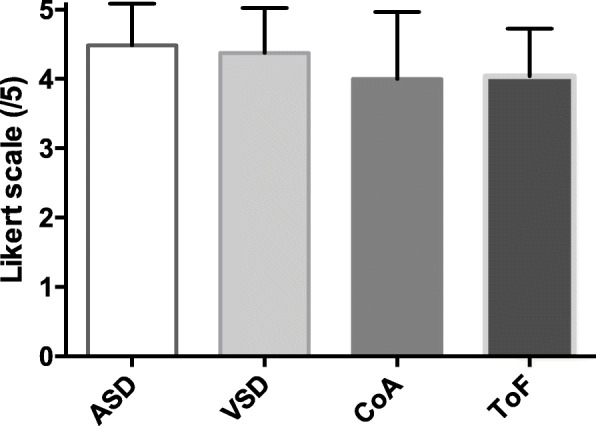
Students’ satisfaction with the 3D models according to the CHD: mean and standard deviation of one 5-point Likert scale item. ASD: Atrial septal defect; CoA: Coarctation of the aorta; CHD: Congenital heart defects; ToF: Tetralogy of Fallot; VSD: Ventricular septal defect

Most of the students strongly agreed that learning CHD through 3D printed models was more useful than through projected images. The score was approximately 4 out of 5 on the 5-point Likert scale regardless of the CHD (Fig. 5).

## Discussion

The main strength of this study is that it evaluated several types of CHD and demonstrated that teaching through 3D printed models improves students’ objective performance in post-lecture scores for each CHD. Moreover, students in the 3D printing group reported a subjective improvement in the understanding of the CHDs, their diagnostic modalities and treatment option with this teaching method compared to a standard lecture. Therefore, the feedback from participants was generally positive and enthusiastic with a higher satisfaction score when 3D printing was involved.

Previous studies reported variable results using different methodologies among medical students or paediatric residents. Based on an objective questionnaire, Su et al. [13] found that 3rd year medical students taught with 3D printed heart models demonstrated a significant improvement in structural conceptualization of VSD compared to a control group. Three-dimensional printed models of vascular rings have also been shown to benefit residents learning about CHD [11]. Nevertheless, Loke et al. found no improvement in factual knowledge acquisition with the use of 3D ToF models for second year residents [14].

Most of the previous studies only reported student opinion rather than objective measures of their learning [10, 15], while only a few used a control group like us [13, 14]. Non-randomization and subjective assessment made the results of these studies less convincing and so strengthen our results. In our study knowledge was enhanced among fifth year medical students in all groups, including the ToF group. We also found no difference regarding the complexity of CHD, while recently Smerling et al. found a correlation [16]. They studied only 45 1st year medical students using a self-reported knowledge survey during a CHD workshop where all students had four different stations (Video, 2D, specimen and 3D), which makes an objective comparison difficult. Nevertheless, they also noted an increase in knowledge regarding all defects.

For decades, heart specimens have been incredibly useful in providing physical 3D samples of heart defects for medical education. Studies suggest no disadvantages to using 3D printed heart models compared to cadaveric specimens, which demonstrates at least equivalent educational outcomes [16, 17]. However, the limited availability, durability and reproducibility of cadaveric heart specimens makes it difficult to spread them and reinforces the interest of 3D printed models to teach CHD. Recently, the Archiving Working Group of the International Society for Nomenclature of Paediatric and Congenital Heart Disease recommends generating 3D digital images of cadaveric heart specimens for printing [18]. From a recent medical educational meta-analysis the 3D printing group had better accuracy and shorter answering time when compared with conventional models (including specimens, plastic products, and 2D anatomical pictures) [19].

The first step towards using 3D printed models in medical education would be to establish local 3D libraries. However, recently some authors have promoted a 3D Heart Library to disseminate validated 3D models through an open-access platform as a peer-reviewed subset of content on the National Institutes of Health 3D Print Exchange [4]. In our experience, models can be easily created from CT scans and 3D echocardiography performed in routine patient follow-up. Several versions of software are available to perform the segmentation and modelling processes. These models are durable and the cost of a printed model is affordable when a 3D printer is already available [7]. The translation of medical imaging data into 3D printed models requires knowledge of anatomy, pathology, imaging physics and engineering concepts related to 3D printing. Therefore, models are most likely to be created by a team.

The impact of teaching with 3D heart models could also be assessed in other situations and has been used to enhance congenital heart critical care via simulation training of multidisciplinary intensive care teams [20].

The recent SARS-CoV-2 pandemic has seen the confirmation of more than 100 million cases at the time of writing, resulting in the imposition of rigorous public health measures such as a quarantine [21, 22]. New interactive forms of virtual teaching have been developed and seem effective and could include virtual-reality or digital 3D PDF files. Beyond the negative effect on students’ mental well-being, technical challenges, confidentiality issues, reduced student engagement, and a lack of assessment have been described [23, 24]. Despite the impact of COVID-19 on medical education, hands-on experience that is provided in a safe environment with 3D printed models should be encouraged.

Limitations of our study include the high specificity of the population, which makes these results difficult to extrapolate to all medical and healthcare professionals. Nonetheless, other researchers have found similar results in populations with highly variable degrees of prior knowledge on CHD [12, 13]. Additionally, we only evaluated the usefulness of 3D printed models for teaching a limited group of simple or moderate CHDs. This is mainly due to our population’s limited prior knowledge of CHD. Therefore, complex CHD such as double outlet right ventricle could not be assessed.

Additionally, we did not test the long-term increase in knowledge to assess knowledge retention. Therefore, we cannot predict whether the positive effects of learning from these models are sustained over a longer period of time.

Finally, 3D printed models do not necessarily provide a good representation of all aspects of heart anatomy and physiology since valve tissue is often poorly recreated. Verbal explanation and guidance remain fundamental for the model.

## Conclusion

3D printed CHD models are a useful resource for teaching in medical schools improving significantly CHD knowledge acquisition for medical students. These findings re-emphasize the role of these models considering the range of CHD severities, and promote the use of this technology in teaching CHD to medical students.

## Supplementary Information

**Additional file 1: S1.** 3D-PDF of tetralogy of Fallot: feel free to crop and navigate into the model.

**Additional file 2: S2.** 3D-PDF of neonatal aortic hypoplasia: feel free to crop and navigate into the model.

**Additional file 3: S3.** 3D-PDF of ventricular septal defect: feel free to crop and navigate into the model.

**Additional file 4: S4.** The post-test questionnaire for tetralogy of Fallot including the objective and subjective questionnaire.

**Additional file 5: S5.** The post-test questionnaire for ventricular septal defect including the objective and subjective questionnaire.

## Data Availability

Additional data and materials can be made available upon request to the corresponding author.

## Abbreviations

2D: Two-dimensional
3D: Three-dimensional
ASD: Atrial septal defect
CoA: Coarctation of the aorta
CHD: Congenital heart defects
CT: Computed tomography
ToF: Tetralogy of Fallot
VSD: Ventricular septal defect
