# Supplementary material for: The usefulness of 3D printed heart models for medical student education in congenital heart disease
Source: BMC Med Educ. 2021 Sep 8;21:480. doi: 10.1186/s12909-021-02917-z (PMC8424617; doi:10.1186/s12909-021-02917-z)
Supplement: Supplementary file 4 — Additional file 4: S4. The post-test questionnaire for tetralogy of Fallot including the objective and subjective questionnaire. [file 12909_2021_2917_MOESM4_ESM.docx]

**S4: The post-test questionnaire for tetralogy of Fallot including the objective and subjective questionnaire.**

| Age : | Sexe : M F |
| --- | --- |

1. Regarding Tetralogy of Fallot (ToF), wich sentence(s) is(are) true?
   1. Ventricular septal defect is generally small
   2. Ventricular septal defect is outlet type
   3. The obstruction is subpulmonary
   4. The aorta is overriding
   5. The deviation of the conal septum is antero - right
2. A newborn is addressed to you for heart murmur, the clinical examination does not find any other abnormality, the weight intake is correct, on the ultrasound you diagnose a Tetralogy of Fallot: wich sentence(s) is(are) true?
   1. This is a cyanotic heart disease
   2. Corrective surgery is an emergency to relieve the obstruction
   3. Sometimes it is necessary to make a palliative surgery of Blalock (systemico-pulmonary anastomosis)
   4. Shunt is usually left to right
   5. Weighting of children with Fallot Tetralogy is often poor
3. An irregular ToF is characterized by?
   1. A nascent interventricular coronary artery from the right coronary artery
   2. A large subaortic ventricular septal defect
   3. Multiple ventricular septal defect
   4. Distal stenosis of the pulmonary arteries
   5. Aortic coarctation
4. Complication(s) of unrepaired ToF is(are)?
   1. Pulmonary overflow
   2. Infectious endocarditis
   3. Tet spells
   4. Heart failure
   5. Severe heart block

Answer these 3 self-rated questionnaires

I understood the congenital heart disease : "Tetralogy of Fallot"

| Strongly disagree | Disagree | Neutral | Agree | Strongly agree |
| --- | --- | --- | --- | --- |
| 1 | 2 | 3 | 4 | 5 |

1. I understood the diagnosis modalities of Tetralogy of Fallot

| Strongly disagree | Disagree | Neutral | Agree | Strongly agree |  |
| --- | --- | --- | --- | --- | --- |
| 1 | 2 | 3 | 4 | 5 | |

1. I understood the different treatment options for Tetralogy of Fallot

| Strongly disagree | Disagree | Neutral | Agree | Strongly agree |  |
| --- | --- | --- | --- | --- | --- |
| 1 | 2 | 3 | 4 | 5 | |
