# Supplementary material for: The usefulness of 3D printed heart models for medical student education in congenital heart disease
Source: BMC Med Educ. 2021 Sep 8;21:480. doi: 10.1186/s12909-021-02917-z (PMC8424617; doi:10.1186/s12909-021-02917-z)
Supplement: Supplementary file 5 — Additional file 5: S5. The post-test questionnaire for ventricular septal defect including the objective and subjective questionnaire. [file 12909_2021_2917_MOESM5_ESM.docx]

**S5: The post-test questionnaire for ventricular septal defect including the objective and subjective questionnaire.**

| Age : | Sex: M F |
| --- | --- |

1. Regarding ventricular septal defect (VSD), wich sentence(s) is(are) true?
   1. Is always located in the muscle part of the inter-ventricular septum
   2. Four type are described regarding the anatomic position into the interventricular septum
   3. Lead to left ventricular dilatation
   4. Some types of VSD are in contact with atrioventricular valves
   5. Some types of VSD are in contact with aortic or pulmonary valves
2. A 2-month-old child is referred to you for heart murmur, the clinical examination does not find any other abnormality, the weight gain is correct, on the ultrasound you find a VSD: wich sentence(s) is(are) true?
   1. The most common VSD are muscular
   2. It is probably small due to the absence of symptoms
   3. Muscular VSD usually close spontaneously
   4. Poor weight may be linked to the VSD
   5. Loud heart murmur is a more severe VSD than without murmur
3. Select among the 5 propositions 4 classical complications of peri-membranous VSD:
   1. Infective endocarditis (jet lesions on the tricuspid valve or septum)
   2. Aortic valve regurgitation by prolapse of a sigmoid
   3. Low infundibular hypertrophy (mid-ventricular barrier)
   4. Pulmonary valve regurgitation by prolapse of a sigmoid
   5. Grade 3 atrio-ventricular block
4. For the treatment of Inter ventricular communications, wich sentence(s) is(are) true?
   1. Medical therapy includes: diuretics, blood transfusion
   2. Curative treatment may be preceded by palliative intervention to reduce pulmonary overflow
   3. Cardiac catheterization closure is the reference technique
   4. Surgery can always repair VSD regardless of its anatomical position
   5. Corrective surgery is possible beyond 5 kg

Answer these 3 self-rated questionnaires

I understood the congenital heart disease : "Ventricular septal defect"

| Strongly disagree | Disagree | Neutral | Agree | Strongly agree |
| --- | --- | --- | --- | --- |
| 1 | 2 | 3 | 4 | 5 |

1. I understood the diagnosis modalities of ventricular septal defect

| Strongly disagree | Disagree | Neutral | Agree | Strongly agree |  |
| --- | --- | --- | --- | --- | --- |
| 1 | 2 | 3 | 4 | 5 | |

1. I understood the different treatment options for ventricular septal defect

| Strongly disagree | Disagree | Neutral | Agree | Strongly agree |  |
| --- | --- | --- | --- | --- | --- |
| 1 | 2 | 3 | 4 | 5 | |
